# Identification of a novel phage depolymerase against ST11 K64 carbapenem-resistant *Klebsiella pneumoniae* and its therapeutic potential

**DOI:** 10.1128/jb.00387-24

**Published:** 2025-03-26

**Authors:** Peini Yang, Bin Shan, Xing Hu, Li Xue, Guibo Song, Pingan He, Xu Yang

**Affiliations:** 1 Department of Clinical Laboratory, The Second Affiliated Hospital of Kunming Medical University 66472 , Kunming, China; 2 Department of Clinical Laboratory, First Affiliated Hospital of Kunming Medical University 36657 , Kunming, China; University of California San Francisco, San Francisco, California, USA

**Keywords:** phage, depolymerase, CRKP, capsular polysaccharide, biofilm

## Abstract

Carbapenem-resistant *Klebsiella pneumoniae* (CRKP) is a clinical pathogen with a high mortality rate, and its clinical management and infection control have become a serious challenge. Phage-encoded depolymerase cleaves the capsular polysaccharide, a major virulence factor of *K. pneumoniae*. This study aimed to identify a phage depolymerase targeting ST11 K64 CRKP, evaluate its antimicrobial activity and therapeutic efficacy, and provide new alternative therapeutic strategies for K64 CRKP. Phages were screened from untreated hospital sewage using clinically isolated CRKP as the host bacterium. The host range, efficiency of plaque formation, optimal multiplicity of infection, adsorption efficiency, and one-step growth curve of phage vB_KpnP_IME1309 were determined by the double-layer agar plate culture method. The morphology of the phage was observed by transmission electron microscopy. Phage nucleic acids were extracted for whole-genome sequencing, and the phage-encoded depolymerase gene ORF37 was amplified by polymerase chain reaction. Next, a recombinant plasmid was constructed to induce depolymerase expression, which was verified using sodium dodecyl sulfate-polyacrylamide gel electrophoresis. *In vitro* bactericidal activity was determined using a combined serum assay, and the anti-*K*. *pneumoniae* biofilm effect of depolymerase was determined by crystal violet staining. Finally, a *Galleria mellonella* larvae infection model was established to investigate the therapeutic effect of depolymerase on larvae *in vivo*. Here, we isolated and characterized a phage vB_KpnP_IME1309 targeting ST11 K64 CRKP, which featured a latent period of 20 min and a burst size of approximately 290 plaque-forming units/cell. It contained 41 predicted open reading frames, of which ORF37 encoded depolymerase. The expressed and purified depolymerase Dep37 cleaved only ST11 K64 CRKP and formed a translucent halo on the agar plate. Dep37 increased the susceptibility of *K. pneumoniae* B1 to serum killing, inhibited CRKP biofilm formation, and degraded mature biofilms. The combination of Dep37 and kanamycin was significantly more effective in treating CRKP biofilms compared to either Dep37 or kanamycin alone. An injection of Dep37 at 5 min and 2 h after the CRKP infection of *Galleria mellonella* larvae increased their survival rates by up to 73% and 53%, respectively. Depolymerase Dep37 may be used as a potential method for capsule typing of *K. pneumoniae*, showing great promise for the development of novel alternative therapeutic strategies against ST11 K64 CRKP.

**IMPORTANCE:**

A novel phage vB_KpnP_IME1309 targeting ST11 K64 carbapenem-resistant *Klebsiella pneumoniae* (CRKP) was isolated and characterized. The ORF37 encoding depolymerase gene of phage vB_KpnP_IME1309 was successfully expressed and purified. Depolymerase increases the susceptibility of CRKP to serum killing, inhibits CRKP biofilm formation, and degrades mature biofilms. The combination of depolymerase and kanamycin is significantly more effective than either depolymerase or kanamycin alone in the treatment of CRKP biofilm. Depolymerase injection at 5 min and 2 h after CRKP infection of *Galleria mellonella* larvae increased the survival rate of larvae by up to 73% and 53%, respectively. Depolymerase Dep37 may be used as a method for the development of novel alternative therapeutic strategies against ST11 K64 CRKP.

## INTRODUCTION

*Klebsiella pneumoniae* is a non-motile, pod-membranous, gram-negative bacterium that is widely present in the environment and capable of colonizing the gastrointestinal and respiratory tracts and the skin of healthy individuals and causing hospital-acquired and community-acquired infections (1–3). With the misuse and abuse of antibiotics, resistant bacteria, such as carbapenem-resistant *Enterobacteriaceae* and carbapenem-resistant *K. pneumoniae* (CRKP), their clinical management and infection control have become a serious challenge (4, 5). According to the February 2023 report of the China Antimicrobial Surveillance Network (www.chinets.com), *K. pneumoniae* has become the second most commonly isolated bacterium in clinics in China, and its resistance rates to meropenem and imipenem (IPM) increased from 2.9% and 3% in 2005 to 24.2% and 22.6% in 2023, respectively. ST11 is the prevalent ST type of CRKP bacteria in China. Gradually, ST11 K64 emerged as the main prevalent subclone type in Chinese clinics instead of ST11 K47 (6, 7). ST11 K64 CRKP has more advantages in capsular polysaccharide (CPS), biofilm, and iron carrier production and is characterized by a wide spectrum of resistance and high pathogenicity (8). Thus, the identification of alternative antibiotic therapies for ST11 K64 CRKP is urgently needed.

*K. pneumoniae* has a variety of virulence factors, such as capsular polysaccharides (CPSs), iron carriers, lipopolysaccharides, pilus and outer membrane proteins, and type 6 secretion system (9). Most *K. pneumoniae* are encapsulated by CPS, which can inhibit the differentiation and function of macrophages, help bacteria escape from phagocytosis, evade the host immune system, and acquire bacterial antibiotic resistance (10–12). CPS makes it easier for bacteria to form biofilms. Therefore, destroying CPS is one of the most important therapeutic strategies against *K. pneumoniae* infection. The CPS is attached to the outside of the outer membrane of the cell and is composed of bacterial CPS synthesis gene clusters conserved on the genome, including *galF*, *orf*2, *wzi*, *wza*, *wzb*, and *wzc* (13). There are more than 80 capsular serotypes of *K. pneumoniae* (14, 15). However, there are currently two main *K. pneumoniae* capsular typing methods: immunology and molecular biology. The traditional serological method is one of the immunological methods. However, the cross-reactivity may also lead to false-positive results (16). Regarding molecular biology methods, polymerase chain reaction (PCR) is used to amplify conserved CPS synthesis genes, such as polymerase *wzy*, outer membrane protein *wzi*, and tyrosine kinase *wzc*, among which the typing based on the *wzi* gene is widely used because of its high specificity and efficiency (17–19). Phage and phage-derived depolymerase were recently reported as new capsular typing methods, whereas depolymerases are more capable of specific recognition than their phages (20, 21).

Phages (bacteriophages), a class of viruses capable of infecting bacteria and replicating themselves, are gaining much attention as targets for antimicrobial strategies (22). The phage genome encodes for the synthesis of a variety of specific enzymes, such as endolysin, depolymerase, and virus particle-associated peptidoglycan hydrolases. Depolymerase is capable of degrading host bacterial CPS, which typically manifests as tail fibers of phages. It promotes the depolymerization of bacterial cells and the adsorption of phage in bacterial biofilms in the form of tail spike protein or free enzyme. Depolymerase has great potential to treat CRKP and the biofilms it produces (23). Phage-encoded depolymerase can be used for capsular typing of *K. pneumoniae*, such as K1 (24), K2, K3, K5 (25), K8, K11, K13, K19 (26), K22, K37 (27), K21, K25, K30/K69, K35, K64, K20, K24, K23 (28), K47 (29), K51, K56, K57 (30), K63, KN1 (31), KN2, KN3, KN4, and KN5. In previous studies, only a few depolymerases, such as ORF38 of phage P510 and ORF41 of SH-KP152410, specifically degraded K64 CRKP, were isolated and characterized (21, 32). Thus, the isolation of a new depolymerase could expand the knowledge on phage depolymerases for K64 CRKP and lay the foundation for using antimicrobial agents to treat clinical CRKP infections.

In this study, we identified and expressed Dep37, a depolymerase from vB_KpnP_IME1309, a novel phage capable of lysing ST11 K64 CRKP, which promotes serum bactericidal susceptibility to ST11 K64 CRKP as well as strong biofilm degradation. In *in vivo* experiments, Dep37 showed promising treatment efficacy against *Galleria mellonella* larvae infected with *K. pneumoniae*. Our results provide preliminary evidence that Dep37 may be a novel alternative approach against ST11 K64 CRKP for the treatment of *K. pneumoniae*.

## MATERIALS AND METHODS

### Collection and identification of *K. pneumoniae* clinical strains

A total of 10 non-replicated *K. pneumoniae* strains, including B1, were collected from patients admitted to a tertiary hospital in Yunnan Province from February to March 2023 and stored at −80°C in a refrigerator via the paper slide method and routinely cultured at 37°C in Luria-Bertani (LB) broth. As previously described, the strains were identified using matrix-assisted laser desorption/ionization time-of-flight mass spectrometry (Bruker Daltonik GmbH, Bremen, Germany). Drug susceptibility was determined with the Vitek 2 compact system (Biomerieux, France), and the results were interpreted according to the Clinical and Laboratory Standards Institute (CLSI) M100-S32 guidelines.

PCR was used to determine the capsular typing of all *K. pneumoniae* isolates as previously described (18). The DNA of *K. pneumoniae* was extracted using a TSINGKE genomic DNA extraction kit, and the *wzi* gene was detected using specific primers (synthesized by the Shanghai Sangon Biotechnology Co., Ltd.). The specific primer sequences were as follows: forward primer (F): GTGCCGCGAGCGCTTTCTATCTTGGTATTCC; reverse primer (R): GAGAGCCACTGGGTTCCAGAAYTTGCACACGC, with a primer size of 580 bp. The amplification conditions were as follows: pre-denaturation, 98°C for 2 min; 35 cycles of denaturation (98°C for 10 s), annealing (63°C for 10 s), extension (72°C for 2 min), and finally final extension (72°C for 5 min). Positive PCR products were sent to Tsingke Biotechnology Co., Ltd. for Sanger sequencing. The sequence results were analyzed using the Pasteur database (https://bigsdb.pasteur.fr/cgi-bin/bigsdb/bigsdb.pl?db=pubmlst_klebsiella_seqdef&page=sequenceQuery).

The genomic DNA of 10 CRKP strains was extracted using a TIANamp Bacterial DNA Kit and sent to Shanghai Majorbio Bio-Pharm Technology Co., Ltd for whole-genome sequencing (WGS) using the Illumina HiSeq 2500 platform. Data were spliced using SPAdes software, and the spliced data were annotated on the RAST website. Based on the Illumina WGS results, the resistance genes in the ST types of the strains were predicted using the online tools ResFinder 4.0 (33) and MLST 2.0 from the Center for Genomic Epidemiology (http://www.genomicepidemiology.org/services/) (34). The WGS results of 10 *K*. *pneumoniae* isolates were uploaded to the Sequence Read Archive (SRA) database (accession no. PRJNA1136661).

### Phage isolation and purification

A novel lytic phage, named vB_KpnP_IME1309, was screened from hospital sewage samples using *K. pneumoniae* B1 of ST11 K64 as the host bacterium as previously described but with slight modification (35). Briefly, the sewage samples were centrifuged for 10 min at 3,500 × *g*, and the supernatant was then filtered through a 0.22 µm filter (Millex-GP Filter Unit; Millipore, USA). The filtered effluent (5 mL) was mixed with 0.5 mL of LB broth containing log-phase *K. pneumoniae* B1, incubated overnight at 37°C, and centrifuged at 3,500 × *g* for 10 min; thereafter, the supernatant was filtered through a 0.22 µm filter. The filtered supernatant was serially diluted in LB and spotted onto *K. pneumoniae* B1-covered agar plates to detect phage spots using the double-layer agar method. The purified isolation process was repeated at least three times until uniform phage spot formation was obtained. Finally, SM buffer containing purified phage was centrifuged for 10 min at 3,000 r/min and filtered through a 0.22 µm syringe-driven filter. The supernatant was added with 10% glycerol and stored at −80°C.

### Determination of phage biological characteristics

#### Observation of phage morphology by electron microscopy

The phage was concentrated and purified as previously described (30). Concentrated vB_KpnP_IME1309 was placed on a copper grid and allowed to stand for 10 min. Excess liquid was aspirated using filter paper, and negative staining was performed with drops of phosphotungstic acid (2%, wt/vol) on a copper grid and allowed to stand for 2 min. The samples were dried for 30 min at room temperature. Images of vB_KpnP_IME1309 were obtained using a transmission electron microscope (JEOL, Tokyo, Japan) at an accelerating voltage of 80 kV.

#### Host range and EOP determination

A spot test was performed to determine the phage’s host range and the recombinant depolymerase’s activity range (36). LB agar plates were covered with agar containing 200 µL of fresh bacterial culture. Phage stock solution (5 µL) was spotted onto the plate; after an overnight incubation at 37°C, the plate was observed for the production of translucent spots.

The efficiency of plating (EOP) of the phage was performed as follows. First, 10 strains of CRKP were inoculated into 3 mL of liquid LB medium each and cultured to the logarithmic stage at 37°C. Next, the phage solution was diluted 10-fold using SM buffer, and 200 µL gradient dilution of phages and bacterial suspension in the logarithmic growth phase were mixed and left at room temperature for 10 min. Then, 3.6 mL of agar was added, and the plate was spread on top of the solid LB plate after sufficient mixing and inverted in a 37°C incubator for overnight cultivation once the agar in the upper layer had solidified. The production of phage plaques was observed the next day. EOP indicates the number of phage plaques produced on the solid LB plate of the target bacterium divided by the number of phage plaques produced on the solid LB plate of the host bacteria (37).

#### Multiplicity of infection, adsorption experiments, and one-step growth curve determination

Multiplicity of infection (MOI) is the ratio of the number of phages to the number of host bacteria when the phages infect the host bacteria (38). Briefly, 0.5 McFarland turbidity (approximately 10^8^ colony-forming units [CFUs]/mL) of *K. pneumoniae* B1 solution was diluted 100-fold to 10^6^ CFU/mL. Then, *K. pneumoniae* B1 was mixed with phage vB_KpnP_IME1309 at MOI values of 0.00001, 0.0001, 0.001, 0.01, 0.1, 1, 10, and 100 and incubated for 15 min at room temperature; 9 mL of LB medium was added to each group, and the mixture was incubated at 37°C for 8 h and centrifuged at 8,000 × *g* for 5 min. The supernatant was filtered through a 0.22 µm disposable filter, and the titer of phage in the supernatant was determined by the double-layer agar method to determine the optimal MOI value of phage. We performed this experiment in triplicate.

Adsorption assays were performed according to the procedure described by Mizuno et al. (39) with slight modifications. The phage solution was mixed with host bacteria at an MOI of 0.01 and then incubated at 37°C. Samples were taken at 0, 2, 4, 6, 8, 10, 12, 14, 16, and 18 min, and the titers of free phages were detected by the double-layer agar method. The adsorption rate of the phage was the percentage of adsorbed bacteriophage potency relative to the total phage potency at each time point. This experiment was performed in triplicate.

A one-step growth curve to determine the latency and burst size of the phage was performed as described by Chakraborty et al. (40) with slight modifications. The host bacterium *K. pneumoniae* B1 was mixed with phage vB_KpnP_IME1309 according to a certain ratio with MOI = 0.00001 (optimal MOI), incubated at 37°C for 5 min, and centrifuged at 12,000 × *g* for 5 min. The supernatant was discarded, the precipitate was resuspended in 1 mL of LB, centrifuged again, and the supernatant was discarded. This procedure was repeated a total of three times to remove unadsorbed phages. The precipitate was suspended in 1 mL LB, transferred to 9 mL LB, and incubated at 37°C in a constant temperature incubator from 0 to 90 min; the samples were taken out every 10 min to determine the phage titer in the cultures using double-layer agar method. This experiment was performed in triplicate. The one-step growth curve of vB_KpnP_IME1309 was plotted using the incubation time as the horizontal coordinate and the logarithm of the phage number as the vertical coordinate. Burst size equaled phage titer at the end of the burst/host bacterial concentration at the beginning of the infection.

#### Stability determination

To determine the thermal and pH stability of vB_KpnP_IME1309, phage suspensions (10^9^ plaque-forming units [PFUs]/mL) were incubated for 1 h at different temperatures (0°C, 4°C, 25°C, 35°C, 45°C, 55°C, 65°C, 75°C, and 85°C) and pH levels (2, 3, 4, 5, 6, 7, 8, 9, 10, 11, 12, and 13) (41). Samples were collected under different conditions, phage spots were determined using the double-layer agar method, and the experiment was repeated three times.

### Phage WGS and bioinformatics analysis

The DNA of vB_KpnP_IME1309 was extracted using a Viral Genome Extraction Kit (Tiangen, Beijing, China) and sent to Shanghai Personalbio Biotechnology Co., Ltd., and the genome was sequenced using an Illumina HiSeq 3000. Genes were assembled, and the results were optimized using SOAP denovo2 software. Data assembly was started after removing adapter contamination and performing data filtering using AdapterRemoval and SOAPec (42, 43). Filtered reads were assembled by SPAdes and A5-miseq to construct scaffolds and overlap clusters (44, 45). GeneMark was used to predict and analyze the open reading frames (ORFs) of the phage genome. Gene annotation was accomplished by searching for functional annotations using the BLAST online tool on the National Center for Biotechnology Information (NCBI) website (https://blast.ncbi.nlm.nih.gov/Blast.cgi), including the Non-Redundant Protein Database, Gene Ontology, Kyoto Encyclopedia of Genes and Genomes, Cluster of Orthologous Groups of proteins, and Swissprot websites (46–49). Predictions of tRNA were made using tRNAScan-SE (50). VirulenceFinder (https://cge.food.dtu.dk/services/VirulenceFinder/) and ResFinder (https://cge.food.dtu.dk/services/ResFinder/) were used to detect the presence of virulence factors and antimicrobial resistance genes in phages, respectively. The genome-annotated sequence of phage vB_KpnP_IME1309 was submitted to NCBI for a sequence number.

To study the evolutionary history of phage vB_KpnP_IME1309 and determine its taxonomic position, Phylogeny.fr (http://www.phylogeny.fr/index.cgi) was used to construct a phylogenetic tree with a “one-button model” of the conserved DNA polymerase of vB_KpnP_IME1309. The Dep37 amino acid sequences were compared against those of other phages using MUSCLE for multiple comparisons, PhyML for phylogeny, and Gblocks to eliminate poorly compared positions and divergent regions. The complete sequences of the phages used in the phylogenetic analysis were downloaded from the GenBank database based on the BLASTp search results.

### Analysis and expression of phage depolymerases

To further analyze the putative tail fiber protein gene (ORF37) of phage vB_KpnP_IME1309, we analyzed the physicochemical properties of the depolymerase ORF37 using ExPasy-ProtParam (https://web.expasy.org/protparam/), Expasy-Protscale analysis (https://www.expasy.org/resources/protscale) for protein hydrophobicity, TMHMM (https://services.healthtech.dtu.dk/services/TMHMM-2.0/), and SignaIP (https://services.healthtech.dtu.dk/services/SignalP-5.0/) analysis of transmembrane structural domains and signaling peptide sequences. In addition, we analyzed the structural/functional domains of Dep37 using different tools, such as BLASTp, HHpred (51), and InterProscan (52). We also analyzed the protein structure using Phyre2 (53) (https://www.sbg.bio.ic.ac.uk/phyre2/html/page.cgi?id=index) and AlphaFold 2 (54).

The tail fiber protein gene (ORF37) of vB_KpnP_IME1309 was predicted to encode a depolymerase. The ORF37 gene was amplified from the DNA of purified phage vB_KpnP_IME1309 using primers. The primer sequences (5′−3′) used were as follows: F: GGAATTCCATATGGAATTCCATGGACCAAGATACTAAAACAATCAT; R: CCGCTCGAGCGGTTAGGCGTTTAGGTAAATACCGG. The 3,054-bp PCR-amplified product was cloned into the *N*-terminal 6× His-tagged pET-28a(+) expression vector through the NdeI/XhoI site. The recombinant plasmid was verified by DNA sequencing, and transformed *Escherichia coli* BL21(DE3) was grown on LB plates containing 50 µg/mL kanamycin. Subsequently, *E. coli* BL21(DE3) was cultured to OD_600_ = 0.5–0.6, and *E. coli* BL21(DE3) was induced using 0.2 mM isopropyl-β-d-thiogalactopyranoside (Sangon Biotech, China) for 15–18 h at 16°C. The mixture was centrifuged, and the supernatant was discarded. The bacterial precipitate was resuspended in Tris-buffered saline, and the target proteins were purified using a Ni-NTA affinity chromatography column after the resuspension solution was added to phenylmethylsulfonyl fluoride at a final concentration of 1 mM and subjected to ultrasonic crushing. After the total protein bound to the Ni-NTA column, it was eluted with a gradient of 20–50 mM imidazole, followed by 100 mM imidazole. Finally, sodium dodecyl sulfate–polyacrylamide gel electrophoresis (SDS-PAGE) was performed, and purified depolymerases were identified using Coomassie Brilliant Blue staining (Thermo Scientific, Waltham, MA, USA).

### Activity of depolymerase Dep37

A gradient dilution of Dep37 was performed to create final concentrations of 100, 10, 1, and 0.1 µg/mL. Serially diluted Dep37 (5 µL) was dropped on plates containing *K. pneumoniae* B1 and observed after incubation at 37°C for 24 and 48 h. The formation of a clear halo on the bacterial plate is a sign of the depolymerase’s antimicrobial activity.

### Bactericidal effect of serum-conjugated depolymerases

The bactericidal effect of serum-conjugated depolymerases was determined as described by Wang et al. (31) with modifications. Serum (5 mL) collected from a healthy volunteer was left at room temperature for 3 h until complete coagulation was achieved and then centrifuged at 5,000 × *g* at low speed for 3 min. The supernatant was retained, while a portion of the serum was aspirated and inactivated at 56°C for 30 min. The experiment was divided into four groups. Each group was supplemented with *Kp*-B1(10 µL) in the logarithmic phase. In Group A, 180 µL of inactivated serum and 10 µL of PBS were added. In Group B, 180 µL of inactivated serum and 10 µL of purified Dep37 were added (100 µg/mL). In Group C, 180 µL of activated serum and 10 µL of PBS were added. In Group D, 180 µL of activated serum and 10 µL of purified Dep37 were added (100 µg/mL). All groups were incubated at 37°C for 6 h. Then, each group was serially diluted, and 100 µL of the dilution was evenly coated on solid LB agar and incubated at 37°C overnight. The number of colonies was calculated. We performed this experiment in triplicate.

### Effectiveness of depolymerase against *K. pneumoniae* biofilms

#### Inhibition of *K. pneumoniae* biofilm formation by depolymerase

In each group of 96-well plates, 100 µL of log-phase *K. pneumoniae* B1, 20 µL of Dep37 (final concentrations of 100, 10, 1, and 0.1 µg/mL), and 80 µL of fresh tryptic soy broth (TSB) medium were added. For the control group, 100 µL *K*. *pneumoniae* B1 was mixed with 100 µL of fresh TSB medium. Three replicate wells were used for each group and incubated at 37°C for 24 h at rest. The supernatant in each well was carefully discarded, and the wells were rinsed twice with 200 µL of PBS. As described by Wu et al. (55) the biofilm content of each well was determined by staining the residue in each with 1% crystal violet and measuring the absorbance value at OD_595_ nm using a Multifunctional enzyme marker (Agilent Technologies, Winooski, VT, USA)

#### Effect of depolymerase on mature *K. pneumoniae* biofilms

*K. pneumoniae* B1 in the logarithmic phase was cultured in 96-well plates at 37°C for 48 h to induce the formation of mature biofilms. The wells were washed three times with PBS. To each, we added 100 µL of Dep37 (final concentrations of 100, 10, 1, and 0.1 µg/mL). Three replicate wells were prepared for each group, while PBS was used as a negative control. The plates were incubated at 37°C for 2 h. The supernatant in each well was carefully discarded, and the plates were washed three times with 200 µL of PBS. The biofilm content of each well was determined by crystal violet staining.

#### Effect of depolymerase combined with antibiotics on *K. pneumoniae* biofilm

The minimum inhibitory concentration (MIC) of kanamycin on the planktonic cells of *K. pneumoniae* B1 was determined. *Klebsiella pneumoniae* 700603 was used as a reference strain. It was found that the MIC of kanamycin was 0.5 µg/mL. Mature biofilms grown for 48 h were treated with various concentrations of kanamycin (0.5, 2, 4, 6, and 8 µg/mL) for 8 h, and a significant decrease in the bacterial counts was observed at a concentration of 4 µg/mL.

To evaluate the effect of combined treatment with Dep37 (10 µg/mL) and antibiotics on biofilm degradation, the experiment was performed as previously described (32). In brief, biofilm formation was induced in 96-well plates for 48 h, and biofilms were washed three times with PBS. Then, the biofilms were divided into six groups: (i) the control group, treated with PBS for 12 h; (ii) the depolymerase group, treated with Dep37 for 12 h; (iii) the kanamycin group, treated with PBS for 3 h and then treated with kanamycin (4 and 8 µg/mL) for 9 h; and (iv) the combination group, treated with Dep37 for 3 h and then treated with kanamycin (4 and 8 µg/mL) for 9 h. After treatment, the biofilms were cleaned and scraped off, and the bacterial culture was diluted to estimate the viable cell count. The bacterial load of the treated biofilm was calculated by determining the viable cell count expressed in log_10_.

#### *G. mellonella* larvae infection model

*G. mellonella* larvae were obtained from Tianjin Huiyuide Biotechnology Co., Ltd. The *G. mellonella* larvae infection model was created with reference to previous studies (26). Briefly, *K. pneumoniae* B1 cultured overnight was incubated in fresh LB at 37°C until the logarithmic period was reached and then centrifuged (8,000 × *g* for 10 min). The bacteria were kept suspended in PBS to a final concentration of 1.5 × 10^6^ CFU/mL. In the infection group, 10 µL of *K. pneumoniae* B1 suspension was inoculated into the left hind limb of the larvae using a syringe. In the treatment group, 10 µL of *K. pneumoniae* B1 suspension was injected initially, followed by 10 µL of Dep37 at 5 min and 2 h later. In the control group, larvae were injected with only 10 µL of PBS. Larvae from all groups were inoculated and kept in the dark at 37°C for 5 days, and the survival rate of each group was recorded daily. Three independent experiments were conducted (*n* = 15 per group). The data from three independent experiments are presented as means.

### Statistical analysis

All statistical analyses were performed using GraphPad Prism (version 10, GraphPad Software, USA). In the experiments evaluating the bactericidal effect of serum-conjugated depolymerases and the effect of depolymerases against *K. pneumoniae* biofilms, data between the two groups of each experiment were analyzed using Student’s *t*-test (*P* < 0.05). In the *G. mellonella* larvae infection model, survival curves were plotted using the Kaplan-Meier method, while the survival analysis was performed using the log-rank Mantel-Cox test. Values of *P* < 0.05 were considered statistically significant.

## RESULTS

### Antibiotic resistance and capsular typing of *K. pneumoniae*

The drug sensitivity analysis showed that 10 strains, including *K. pneumoniae* B1, were resistant to a variety of antibiotics, including meropenem, IPM, ertapenem, amikacin, cefuroxime, Cefepime, piperacillin, and ciprofloxacin. All the 10 strains belonged to CRKP. These strains were categorized into two ST strains (ST231 and ST11) and three serotypes (K51, K47, and K64).

### Isolation, host range, and EOP of phage vB_KpnP_IME1309

**Fig 1:**
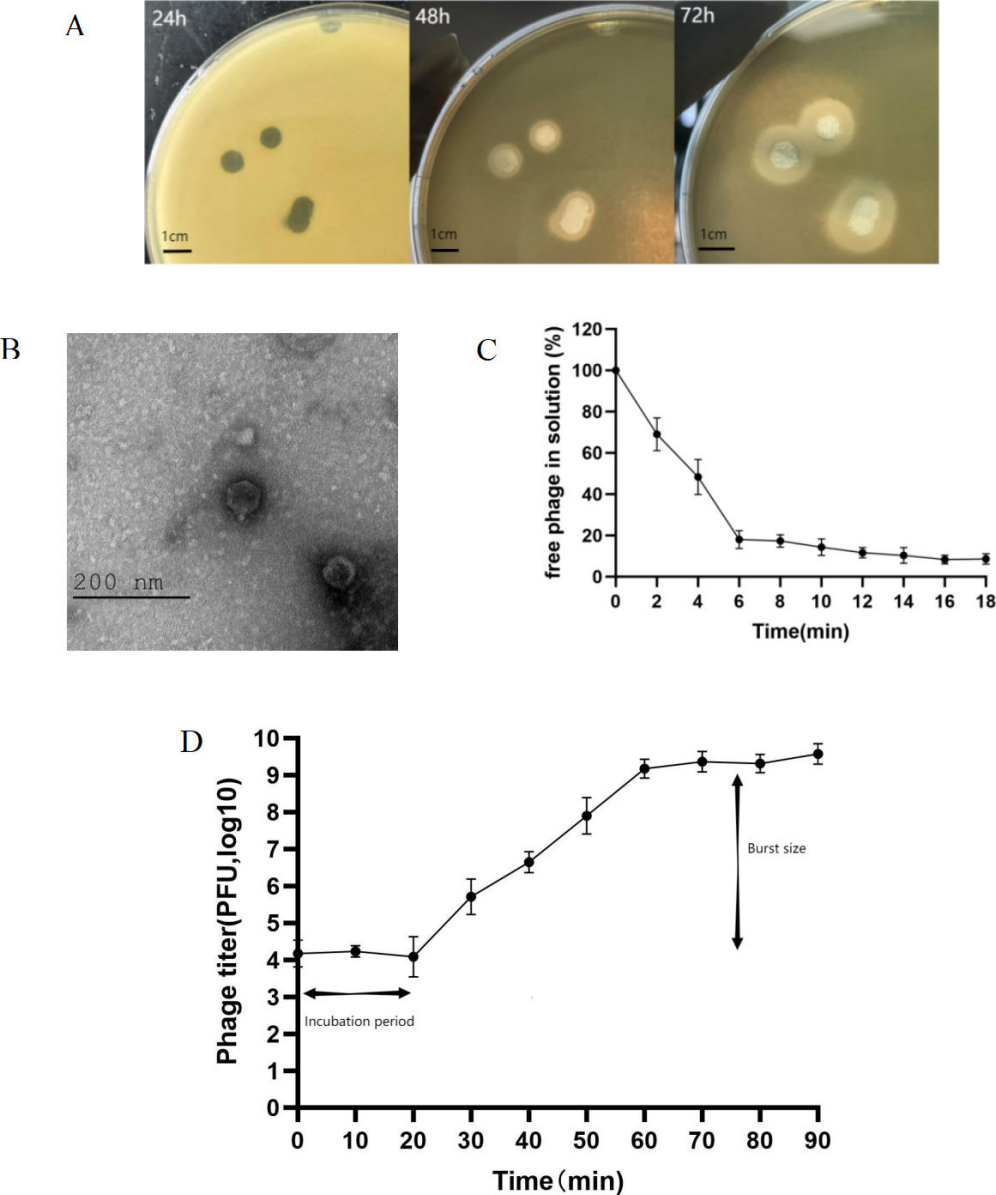
Phage spots, transmission electron microscopy (TEM), adsorption experiments, and one-step growth curves of phage vB_KpnP_IME1309. (**A**) Phage spots of phage vB_KpnP_IME1309 at 24, 48, and 72 h of incubation. (**B**) TEM of phage vB_KpnP_IME1309. (**C**) Adsorption experiments of phage vB_KpnP_IME1309. (**D**) Phage one-step growth curve. Burst size is equal to the phage titer at the end of the outbreak/host bacterial concentration at the beginning of the infection. Error bars indicate mean ± standard deviation.

Using *K. pneumoniae* B1 as the host bacterium, a new phage, vB_KpnP_IME1309, was isolated from sewage collected from hospitals. Phage vB_KpnP_IME1309 was able to form clear phage plaques with diameters of 4.00–7.00 mm when spread on agar plates containing *K. pneumoniae* B1 and incubated at 37°C for 24 h. However, no obvious halos were observed (Fig. 1A). After successive 48 and 72 h incubations, the plaques began to show halos, and the halos increased in size over time, suggesting that phage vB_KpnP_IME1309 may encode a depolymerase.

The host range of phage vB_KpnP_IME1309 was tested using the 10 clinically sourced CRKP described above. Phage vB_KpnP_IME1309 had a narrow host range and could only lyse ST11 K64 CRKP (Table 1), with a lysis rate of 80% (8/10). These CRKP strains were sensitive to vB_KpnP_IME1309 and had EOP values of 0.04–1 (Table 1).

**TABLE 1:** Host range of phage vB_KpnP_IME1309 and depolymerase Dep37^*a*^

Strains	Serotype	ST	Carbapenemase gene	Source	Phage vB_KpnP_IME1309	EOP	K64-Dep37
*K. pneumoniae* B1	K64	ST11	*bla*_KPC-2_	Sputum	＋	1.00	＋
*K. pneumoniae* B2	K64	ST11	*bla*_KPC-2_	Drainage fluid	＋	0.20	＋
*K. pneumoniae* B3	K64	ST11	*bla*_KPC-2_	Urine	＋	0.10	＋
*K. pneumoniae* B4	K51	ST231	–^*b*^	Wound secretion	−	−	−
*K. pneumoniae* B5	K64	ST11	*bla*_KPC-2_	Sputum	＋	0.80	＋
*K. pneumoniae* B6	K47	ST11	*bla*_KPC-2_	Feces	−	−	–
*K. pneumoniae* B7	K64	ST11	*bla*_KPC-2_	Sputum	＋	0.05	＋
*K. pneumoniae* B8	K64	ST11	*bla*_KPC-2_	Sputum	＋	0.04	＋
*K. pneumoniae* B9	K64	ST11	*bla*_KPC-2_	Sputum	＋	0.08	＋
*K. pneumoniae* B11	K64	ST11	*bla*_KPC-2_	Sputum	＋	0.30	＋

^*a*^ The lytic activity of phage vB_KpnP_IME1309 and depolymerase Dep37 against 10 CRKP strains was detected using the spot method (−, no lysis; +, showing plaques or halos).

^*b*^ -, No relevant gene was detected.

### Morphology and MOI of phage vB_KpnP_IME1309

Measurements based on transmission electron microscopy (Fig. 1B) revealed that phage vB_KpnP_IME1309 had a 20-sided head (diameter, 50.52 nm) and a tail (length, 12.87 nm; width, 7.28 nm). It belongs to the *Podoviridae* family of phages. Phage vB_KpnP_IME1309 had an optimal MOI of 0.00001. The progeny phage produced at this time was 3.47 × 10^9^ PFU/mL (Table 2). In the adsorption experiments (Fig. 1C), when the phage vB_KpnP_IME1309 and host bacteria were incubated together for 6 min, approximately 82% of the phages were adsorbed onto the host bacteria; when the phage and host bacteria were incubated together for 16 min, 92% of the phages were adsorbed and stabilized. The one-step growth curve (Fig. 1D) showed that vB_KpnP_IME1309 had an incubation period of 20 min and a burst size of 290 PFU/cell.

**TABLE 2:** Determination of MOI of phage vB_KpnP_IME1309

MOI	Phage (PFU/mL)	Host (CFU/mL)	Product (PFU/mL)
100	10^8^	10^6^	5.43 × 10^7^
10	10^7^	10^6^	2.00 × 10^8^
1	10^6^	10^6^	3.60 × 10^8^
0.1	10^5^	10^6^	2.10 × 10^8^
0.01	10^4^	10^6^	1.60 × 10^8^
0.001	10^3^	10^6^	3.80 × 10^8^
0.0001	10^2^	10^6^	5.93 × 10^8^
0.00001	10^1^	10^6^	3.47 × 10^9^

### Phage vB_KpnP_IME1309 stability

**Fig 2:**
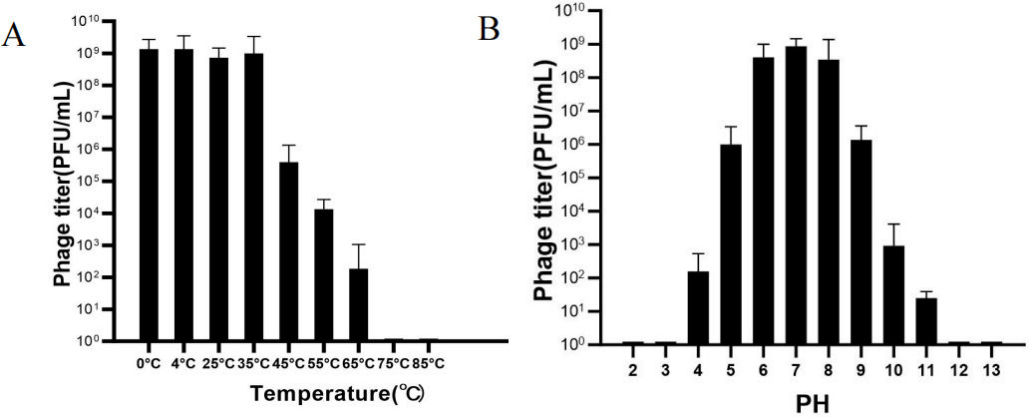
Effect of temperature and pH on depolymerase stability. (**A**) Exposure of the depolymerase to 0°C–85°C resulted in loss of Dep37 activity at temperatures above 65°C. (**B**) Dissolution of the depolymerase in buffers with pH values ranging from 2 to 13. We observed that the optimum enzyme activity for depolymerase was between pH 4 and 11.

As shown in Fig. 2A, phage vB_KpnP_IME1309 had high activity at 0°C–35°C but began to decrease at >45°C. The phage was completely inactivated after 1 h at ≥75°C. As shown in Fig. 2B, phage vB_KpnP_IME1309 was active at pH 4–11 and lost activity at a pH of 1–3 or >12.

### Bioinformatics analysis of phage vB_KpnP_IME1309 genome

**Fig 3:**
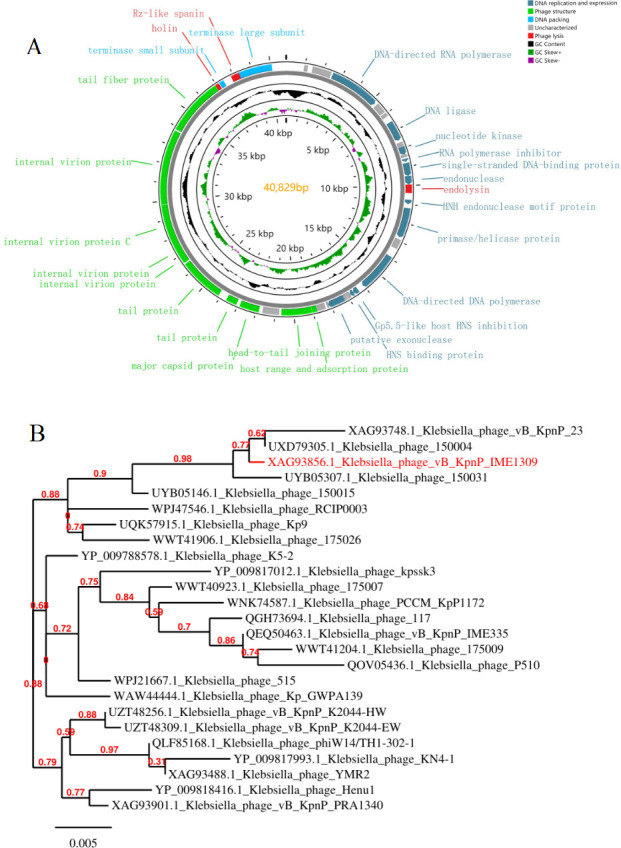
Genetic map of phage vB_KpnP_IME1309 and phylogenetic analysis of phage DNA polymerases of the genus *Przondovirus*. (**A**) Genetic map of phage vB_KpnP_IME1309. Different colored arrows indicate predicted coding sequences (CDSs) encoding different functions: red, lysis proteins; gray, proteins of unknown function; dark blue, DNA replication and expression; green, phage structure; and light blue, DNA packaging. (**B**) The phylogenetic tree was inferred in “one-click mode” by using Phylogeny. Fr (http://www.phylogeny.fr/index.cgi) in “one-click mode” for inference, MUSCLE for multiple matching, PhyML for phylogeny, and Gblocks for the elimination of poorly matched positions and divergent regions. The login number is displayed in front of the phage name. Phage VB_KpnP_IME1309 is labeled in red.

The genome of phage vB_KpnP_IME1309 was registered in the NCBI GenBank database (accession no. PP734006.1). The genome of phage vB_KpnP_IME1309 shows that it is a linear dsDNA molecule with a length of 40,829 bp and guanine–cytosine content of 52.64% (25.42% adenine, 24.86% cytosine, 27.78% guanine, and 21.94% thymine). The analysis showed that phage vB_KpnP_IME1309 had 41 ORFs (Fig. 3A), with a total length of 34,935 bp (85.56% coding percentage); the mean ORF size was 852.07 bp. The proteins encoded by the vB_KpnP_IME1309 genome were categorized into the following groups based on their predicted functions: phage replication and expression (12 ORFs), phage structure (10 ORFs), DNA packaging (two ORFs), phage lysis (three ORFs), and phage unknown function proteins (14 ORFs). The detailed annotation information is provided in Table S1. No pathogenic virulence factors, antibiotic resistance genes, lysogeny genes, or tRNAs were found in its genome, indicating its safety for therapeutic applications.

According to the BLASTn analysis, phage vB_KpnP_IME1309 shared high nucleotide similarity with *Klebsiella* phages 175004 (PP357454.1), 150007 (OP103917.1), 150021 (OP103921.1), 150004 (OP045496.1), and P06 (OR387547.1), with 93% coverage and 95.57%–97.34% identity. A phylogenetic analysis (Fig. 3B), based on the DNA polymerase-encoding gene sequence of phage vB_KpnP_IME1309, indicated that it belongs to the genus *Przondovirus*. As indicated by the species delimitation criteria developed by the International Committee on Taxonomy of Viruses (56), the primary species delimitation criterion for bacterial and archaeal viruses was set at 95% overall DNA sequence homology. Phage vB_KpnP_IME1309 has the highest nucleotide similarity to *Klebsiella* phage 175004, which has an overall DNA sequence homology of 90.52% (Table S2). Therefore, phage vB_KpnP_IME1309 should be considered a new member of the genus *Przondovirus*.

### Sequence analysis of depolymerase

ORF37 in phage vB_KpnP_IME1309 was predicted to be a tail fiber protein (TFP) with a possible depolymerase function and named Dep37. ExPasy-ProtParam predicted the physicochemical properties of Dep37, the molecular weight of which was 110.06 kDa and the theoretical isoelectric point was 5.65. Dep37 has 1,017 amino acids, including 93 negatively charged and 83 positively charged. The half-life was >10 h in *E. coli*, while its instability coefficient was 19.06 (<40). The Expasy-Protscale protein hydrophobicity analysis showed that the maximum and minimum values of Dep37 hydrophobicity were 2.467 and −2.289, respectively. TMHMM and SignaIP did not predict that Dep37 has transmembrane structural domains or signal peptide sequences.

**Fig 4:**
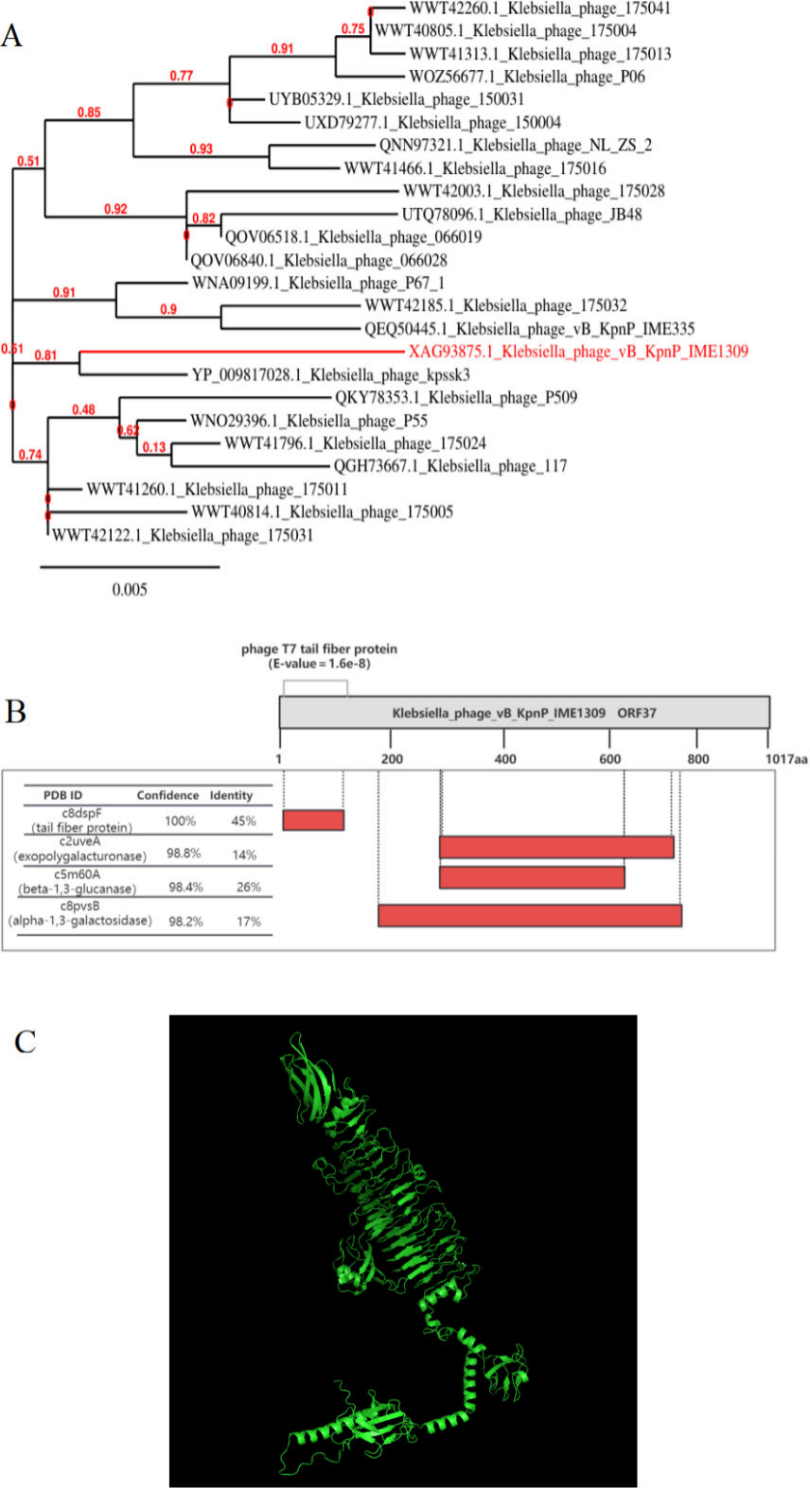
Phylogenetic tree of phage vB_KpnP_IME1309 and structural/functional domain analysis of ORF37. (**A**) A phylogenetic tree based on phage ORF37 was constructed by using protein sequence comparisons of different phages. (**B**) As indicated by Phyre2 results, depolymerase Dep37 is a 1,017 aa protein with several conserved structural domains: phage T7 tail fibronectin (7–155), α-1,3-galactosidase (180–763), β-1,3-glucanase (275–648), and exopolygalacturonase (274–777). (**C**) Three-dimensional structures of Dep37 analyzed by using AlphaFold 2.

A BLASTp sequence comparison showed high homology (coverage, 100%; identity, 98.82%) between Dep37 and the TFP of *Klebsiella* phage kpssk3 (accession no. YP_009817028.1), and both phages were located on the same branch of the phylogenetic tree (Fig. 4A). A Phyre2 server analysis of the Dep37 protein structure showed that it contains a conserved *N*-terminal phage_T7 structural domain (Fig. 4B), similar to that of alpha-1,3-galactosidase (confidence, 98.2%; identity, 17%), beta-1,3-glucanase (confidence, 98.4%; identity, 26%), and exopolygalacturonase (confidence, 98.8%; identity, 14%) with varying degrees of confidence and similarity. The domain analysis of HHpred and InterProscan showed that Dep37 possesses the tail fiber protein domain and tail spike domain (Table 3). In addition, we showed the structure of the protein by the AlphaFold 2 website. The protein structure contained more β-helix (Fig. 4C).

**TABLE 3:** The different domain analyses of Dep37

Tool				
NCBI blastp	Homolog (accession no.)		Query coverage	Sequence identity
	Tail fiber protein of *Klebsiella* phage kpssk3 (YP_009817028.1）		100%	98.82%
	Tail fiber protein of *Klebsiella* phage P55 (WNO29396.1）		100%	98.43%
	Tail fiber protein of *Klebsiella* phage vB_KpnP_IME335 (QEQ50445.1）		100%	98.33%
**HHpred**	**Family**	**Identifier**	**Motif (aa)**	
	Tail fiber protein	A0A2K9VGS2_9CAUD	1–1,017	
**InterProscan**	**Family**	**Identifier**	**Motif (aa)**	
	Phage T7 tail fiber protein	IPR005604	6–152	
	Tail spike TSP1/Gp66	IPR040775	187–241	

### Dep37 expression and activity assay

**Fig 5:**
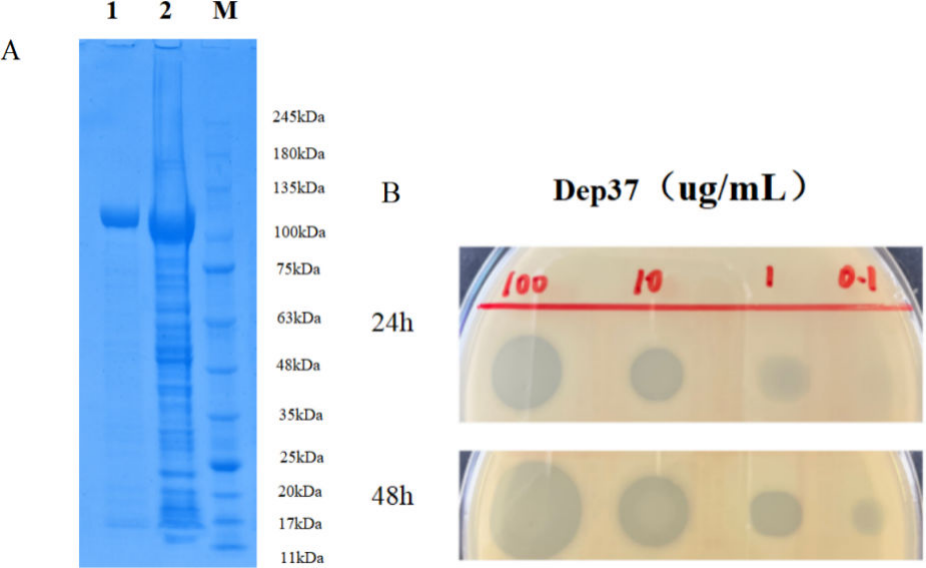
Expression of depolymerase and its activity. (**A**) Expressed Dep37 was electrophoresed by SDS-PAGE. Lane 1 is the purified depolymerase, lane 2 is the total protein lysate, and M represents molecular marker. (**B**) Purified depolymerase Dep37 (100, 10, 1, and 0.1 µg/mL) was dropped on the lawn of a double agar plate containing the host bacterium *Kp*-B1. The plates were incubated at 37°C for 24 and 48 h, respectively, for observation.

We successfully expressed and purified Dep37 from phage vB_KpnP_IME1309. As expected, an SDS-PAGE analysis revealed that its protein was approximately 110 kDa long (Fig. 5A). Serial dilutions of Dep37 subsequently dropped onto plates containing *Kp*-B1 bacteria showed that the minimum concentration of Dep37 for halo formation was 0.1 µg/mL (Fig. 5B). Consistent with phage vB_KpnP_IME1309, the host range showed that Dep37 was able to form clear halos on only eight plates containing K64 CRKP; thus, it can be used for the specific identification of the K64 serotype *K. pneumoniae*.

### Bactericidal effect of serum-conjugated depolymerases

**Fig 6:**
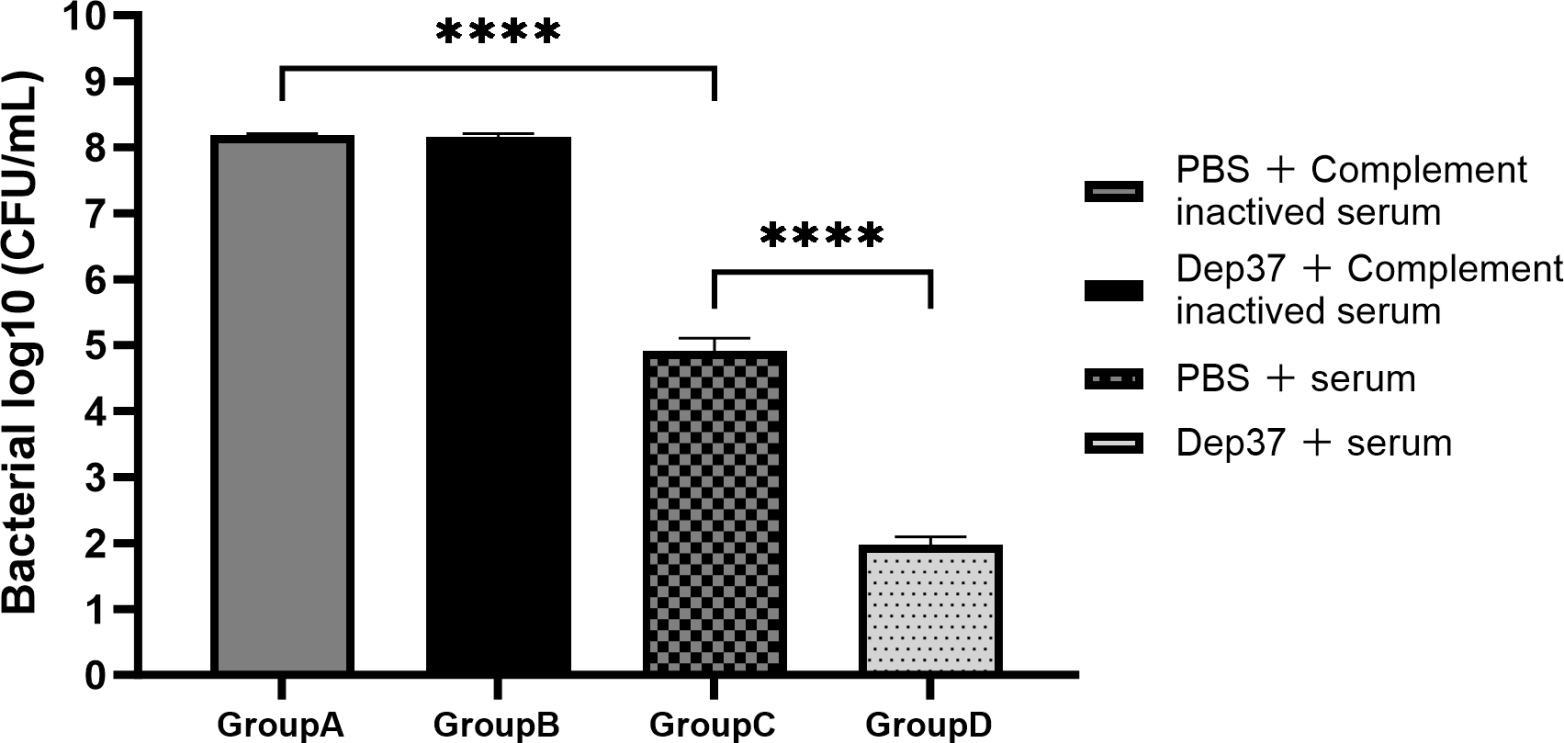
Bactericidal effect of serum combined with depolymerase. After treatment with depolymerase (100 µg/mL), serum, or their combination for 6 h, the number of bacteria was counted on agar plates. The results showed that bacteria were sensitive to human serum, but treatment with depolymerase alone did not kill bacteria. The depolymerase treatment increased the killing effect of serum on bacteria. Three experiments were performed, and Student’s *t*-test was used for bacterial counts between the two groups (*P* < 0.05).

As shown in Fig. 6, after a 6 h incubation, the bacterial counts of groups A, B, C, and D were 8.186, 8.164, 4.910, and 1.976 log CFU/mL, respectively. Compared to group C (PBS combined with serum group), the bacterial counts of group D (Dep37 combined with serum group) were reduced by 2.934 log CFU/mL. However, when Dep37 and inactivated serum were combined, no significant reduction in the number of bacteria was observed. These results suggest that depolymerase enhances the complement-mediated bactericidal effect of the serum.

**Fig 7:**
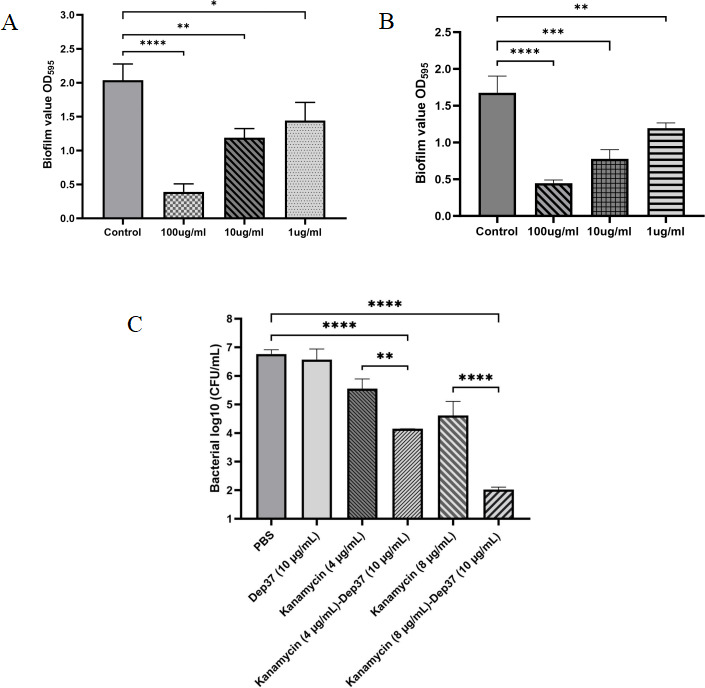
Antibiofilm and antibacterial activity of Dep37. (**A**) Dep42 inhibited the formation of biofilms. Dep37 at 1, 10, and 100 µg/mL was incubated with *K. pneumoniae* B1 in 96-well plates for 24 h. The residual biofilm was assessed by crystal violet staining, and the absorbance was measured at 595 nm. (**B**) Dep37 disrupted the mature biofilm. Biofilm formation of *K. pneumoniae* B1 was induced in 96-well plates for 48 h, and then, the biofilm was treated with depolymerase at 1, 10, and 100 µg/mL for 2 h. The residual biofilm was assessed by crystal violet staining. (**C**) Effect of Dep37 combined with antibiotics on bacterial biofilm. Biofilm formation of *K. pneumoniae* B1 was induced in 96-well plates for 48 h, and then, the biofilm was treated with Dep37 (10 µg/mL), kanamycin (4 and 8 µg/mL), or Dep42 (10 µg/mL), followed by kanamycin (8 µg/mL) as indicated. The treatment groups with PBS were used as negative controls. The viable bacterial count was determined on LB agar plates. *P < 0.05, **P < 0.01, ***P < 0.001, and ****P < 0.0001.

### Efficacy of depolymerase against *K. pneumoniae* biofilms

To evaluate the ability of Dep37 to inhibit biofilm formation and disintegrate mature biofilms, different concentrations were added before and after biofilm formation by *K. pneumoniae* B1. The result showed that Dep37 at 100, 10, and 1 µg/mL concentrations could significantly inhibit biofilm formation and disintegrate mature biofilm in a dose-dependent manner (Fig. 7A and B)

### Effect of depolymerase combined with antibiotics against *K. pneumoniae* biofilms

Depolymerase degraded the bacterial surface CPS but had no bactericidal effect; therefore, it was used in conjunction with antibiotics to evaluate its effect on CRKP biofilms. When combined with antibiotics, Dep37 increased bacterial susceptibility to kanamycin (Fig. 7C). Treatment of mature biofilms with Dep37 alone (10 µg/mL) did not cause significant changes in bacterial counts. However, when Dep37 (10 µg/mL) was used in combination with 4 and 8 µg/mL of kanamycin, the bacterial counts of mature biofilms were reduced by about 2.62 and 4.74 log CFU/mL, respectively.

### Depolymerase improves survival of the *G. mellonella* larvae infection model

**Fig 8:**
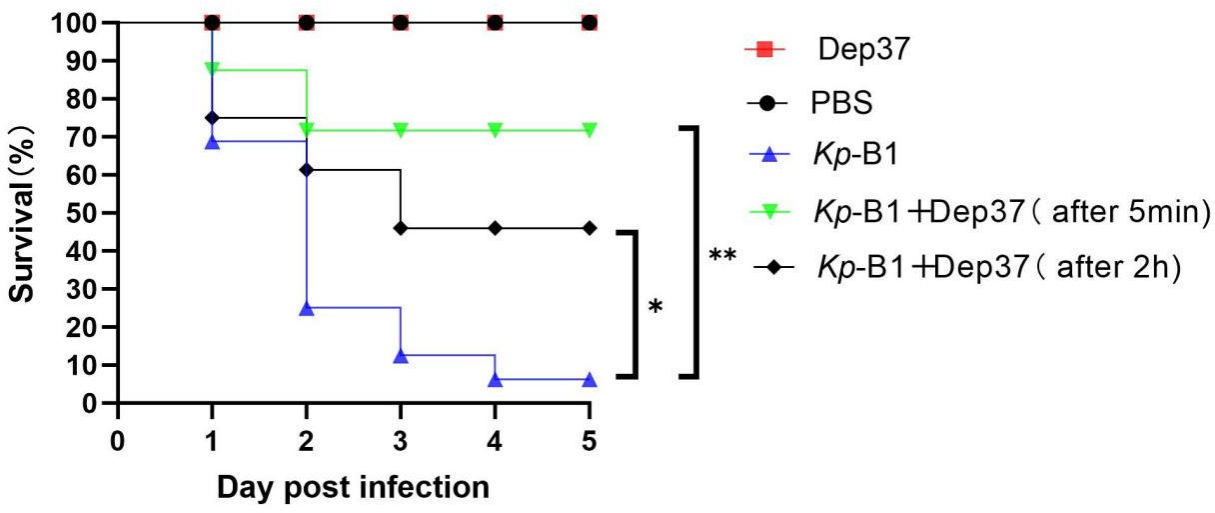
Kaplan-Meier survival curves of *Galleria mellonella* larvae infected with *K. pneumoniae* B1. Experiments were set up in which *Galleria mellonella* larvae were used as controls by being injected only with PBS, and Dep37 was used as the control group. Dep37 was injected 5 min and 2 h after *K. pneumoniae* B1 injection as the treatment groups. The survival analysis was performed using the log-rank Mantel-Cox test.

We evaluated the therapeutic efficacy of Dep37 in the *G. mellonella* larval infection model. No mortality was observed in the larvae treated with PBS or Dep37 within 5 days (Fig. 8). Among the *K. pneumoniae* B1-infected group, approximately 80% (12/15) of the larvae died within 2 days, while 100% died within 4 days. Compared to the infected group, a single injection of Dep37 at 5 min after the *K. pneumoniae* B1 injection in the treatment group increased the survival rate of the larvae to 73% (11/15), whereas a single injection of Dep37 at 2 h after the *K. pneumoniae* B1 injection increased the survival rate to only 53% (8/15).

## DISCUSSION

In recent years, with the misuse of antibiotics and inadequate development of new antibacterial treatments, microorganisms have acquired anti-microbial resistance, leading to a pressing global public health threat. The World Health Organization has estimated that the number of deaths due to drug-resistant bacterial infections will increase to 10 million by 2050 (57). *K. pneumoniae*, as one of the ESKAPE pathogens (i.e., *Enterococcus faecium*, *Staphylococcus aureus*, *K. pneumoniae*, *Acinetobacter baumannii*, *Pseudomonas aeruginosa*, and *Enterobacter* species) members (58), can develop resistance to a variety of antimicrobial drugs via multiple mechanisms. For example, CRKP infections can lead to treatment failure, prolonged hospitalization, and increased mortality (59). Therefore, new sensitive antimicrobial agents and therapeutic strategies for CRKP infections are urgently required.

Here, we isolated a phage strain from sewage water that lyses ST11 K64 CRKP. The BLASTn comparison showed that the whole genome of vB_KpnP_IME1309 had the highest genome coverage of 93%, whereas its overall DNA sequence homology was <95% compared to that of any phage in the NCBI bacteriophage library and belonged to the *Przondovirus* genus of novel phages. Phage stability is closely related to its storage, transportation, and treatment. Only high-stability phages have the potential for successful application as alternative antibacterial agents (60). Biological characterization of the phage vB_KpnP_IME1309 showed that it operates optimally at 45°C and a pH of 4–11. The infection efficiency of the phage was high, with an optimal infection complex of only 0.00001, indicating that the phage proliferated most efficiently at this time and produced the largest number of progeny. The optimal MOI culture was often selected when preparing a large number of phages, thus reducing the production cost and increasing the profit. Phages with short latencies and large bursts can produce progeny more quickly and in greater numbers, which is favorable for rapid and massive phage amplification. Phage vB_KpnP_IME1309 had a latency of 20 min and a large burst of 290 PFU/cell. Combined with these biological characteristics, phage vB_KpnP_IME1309 is a promising potential therapeutic agent for K64 CRKP.

It is worth noting that phage vB_KpnP_IME1309 formed phage plaques (diameter, 4.00–7.00 mm) without a distinct halo on double-layer LB plates after overnight incubation, and the halo increased gradually with incubation time, a finding that is similar to that of the previously reported phage P13 that targets ST11 K47 CRKP (61). The reasons for this phenomenon are diverse, as the phage spot characteristics depend on its adsorption rate, lysis time, burst size, and morphology. Phenotypically, phages vB_KpnP_IME1309 and P13, both of the *Podoviridae* family, showed similar characteristics, with adsorption rates of 82% and 95%, respectively, within 6 min. A single-step growth curve experiment indicated a latency of 20 min. The burst sizes of phages vB_KpnP_IME1309 and P13 were 290 and 167 PFU/cell, respectively. According to the genotypic analysis, the burst size and lysis time of phages are controlled by the holin–endolysin system. When phages infect gram-negative bacteria, lytic proteins gradually accumulate. Thereafter, holin forms a transmembrane pore in the inner membrane of the host, which enables the endolysin to reach the peptidoglycan (PG) layer of the cell wall from the cytoplasm, thus cleaving the PG. Finally, the outer membrane of the host is disrupted by spanins, leading to bacterial lysis of the bacterium (62).

Although the genes encoding holin and endolysin are generally adjacent in the phage genome, the genes encoding holin (ORF38) and endolysin (ORF13) are distinct in the vB_KpnP_IME1309 genome, and this unique genetic structure is also present in phage P13. Further comparison of the amino acid sequences of holin and endolysin between vB_KpnP_IME1309 and P13 revealed that the two phage strains had high homology of holin (coverage, 98%; identity, 98.4%), with the only difference being the last amino acid (lysine versus arginine). Moreover, the endolysins of the two phages shared high homology (coverage, 100%; identity, 98.68%), differing at only two amino acid sites. Thus, the high similarity between phages vB_KpnP_IME1309 and P13 in the holin–endolysin system may be one reason for the appearance of similar phages. In addition, the genomes of phage vB_KpnP_IME1309 and the *Przondovirus* genus contain Rz-like spanin (Rz-like lysis protein), which serves as a signaling anchor for connecting and mediating fusion of the inner and outer membranes of the host, leading to complete host cleavage (63).

Although phages can persistently multiply and infect their bacterial hosts *in vivo*, their quality as biological agents is not easily controlled, and significant safety issues related to their clinical application require addressing (64). In addition to the direct application of intact phages, phage-encoded CPS depolymerase can specifically degrade bacterial polysaccharides. Phage-carried enzymes, which are safer, easier to produce, and easier to purify than phages, are considered promising therapeutic agents.

Phage vB_KpnP_IME1309 was able to form a halo after 48 h of incubation on a double-layer agar plate and increase the halo with the incubation time, suggesting that it may encode depolymerase. Unlike phages, depolymerases do not have cleavage activity or kill host bacteria; they are mainly located within receptor-binding proteins, which help to initiate phage infection by recognizing and degrading bacterial CPS. At the structural level, depolymerases usually have a modular structure consisting of three structural domains: conserved *N*-terminal, variable central, and *C*-terminal (65). The central structural domain is capable of forming a homotrimeric structure containing a β-helix that helps with host recognition and enzymatic activity. ORF37 of phage vB_KpnP_IME1309 encodes TFP with a central structural domain that is similar to glycosidases, glucanases, and pectin cleavage enzymes and is predicted to be a depolymerase. Dep37 identified in this study showed similar features to previously reported depolymerases (ORF38 of phage P510 and ORF41 of SH-KP152410), all of which were from phages of the *Podoviridae* family according to the BLASTp comparison. Moreover, we found a high similarity of Dep37 to ORF38 (coverage, 100%; identity, 97.25%) of phage P510 and ORF41 (coverage, 100%; identity, 97.15%) of SH-KP152410. These three depolymerases were capable of degrading only the CPS of K64 CRKP. We successfully expressed and purified Dep37 from phage vB_KpnP_IME1309. Consistent with vB_KpnP_IME1309, Dep37 showed specific activity against only K64 CRKP cells, suggesting its potential role in capsular typing. We performed *in vivo* and *in vitro* experiments to further explore the role of Dep37 in treating CRKP infections.

Bacterial biofilms are complex microbial communities that attach to biological and non-microbial surfaces and form under the wrapping of extracellular polymeric substrates composed of a large number of polysaccharides, extracellular DNA, lipids, and proteins that are self-produced by bacteria (66). Bacterial biofilms can help bacteria defend themselves against unfavorable external environmental conditions through a variety of mechanisms to enhance bacterial drug resistance. Our results show that Dep37 inhibits CRKP biofilm production and can degrade mature biofilms. However, depolymerase does not kill the host bacteria; it is associated with only CPS disruption. The use of phage-encoded depolymerases as the sole treatment for disrupting bacterial biofilms is insufficient. A combination of depolymerases and other antimicrobial agents will have better bactericidal effects, such as antibiotics, phages, chemicals, natural compounds, and disinfectants (67, 68). For example, the mechanism of the synergistic effect between phage depolymerases and antibiotics is that when depolymerases remove CPS, the arrangement of bacteria in the biofilm becomes dispersed, thus enhancing the ability of antibiotics to penetrate the biofilm. Latka and Drulis-Kawa (69) reported that the combined use of depolymerase with ciprofloxacin and phages was effective against biofilms of multidrug-resistant *K. pneumoniae*. Wu et al. (55) showed that phage depolymerase Dep42 could enhance the activity of polymyxin against *K. pneumoniae* biofilms. In our study, the application of depolymerase with kanamycin to CRKP biofilms had a synergistic effect that enhanced its bactericidal effect, suggesting that depolymerase can be an adjunctive antimicrobial membrane agent to antibiotics. In the review by Guo et al. (70), some antibiotics have a synergistic effect with phage depolymerase, such as ciprofloxacin, colistin, and polymyxin. While some antibiotics, such as imipenem and amikacin, have no synergistic effect with phage depolymerase. Therefore, it is necessary to select appropriate antibiotics for synergistic depolymerase degradation of biofilms.

Phage-encoded depolymerases have recently been shown to effectively treat *K. pneumoniae*-infected mice and *G. mellonella* larvae. In our study, no cases of mortality were observed among the larvae of the PBS- or Dep37-injected groups within 5 days, demonstrating the non-toxicity of depolymerase. A single injection of Dep37 at 5 min or 2 h after the injection of *K. pneumoniae* B1 increased the survival rate of *G. mellonella* larvae to 73% and 53%, respectively, demonstrating its effective therapeutic effect.

This study has several limitations. First, bacteriophage vB_KpnP_IME1309 and its depolymerase have a limited host range. We only collected CRKP strains of three capsular types, and the ability of phage and its depolymerase to lyse CRKP of other capsular types is unknown. Second, although we demonstrated the safety and efficacy of vB_KpnP_IME1309 through a *G. mellonella* larvae infection model, it needs to be further validated at the clinical level. Finally, there are complex interactions between phage depolymerases and host bacteria. To fully understand the bactericidal effects of phage depolymerases, their detailed mechanisms of action need to be investigated through more experiments.

### Conclusion

In conclusion, we have identified and expressed Dep37 from a novel phage targeting K64 CRKP. Our results indicated that Dep37 possesses high degradation activity against CPS that can inhibit CRKP biofilm production and degrade already formed biofilms. We also demonstrated the synergistic therapeutic effect of Dep37 and kanamycin against *K. pneumoniae* biofilms. In addition, Dep37 showed effective therapeutic effects in the *G. mellonella* larval model of K64 CRKP infection. These results suggest that Dep37 is a promising candidate for the control and treatment of CRKP infections.

## SUPPLEMENTAL MATERIAL

Supplemental material jb.00387-24-s0001.docx Annotation of phage vB_KpnP_IME1309 sequence.

Supplemental file jb.00387-24-s0002.xlsx The data of antibiotic resistance of *K. pneumoniae*.

ASM does not own the copyrights to Supplemental Material that may be linked to, or accessed through, an article. The authors have granted ASM a non-exclusive, world-wide license to publish the Supplemental Material files. Please contact the corresponding author directly for reuse.
