# Supplementary material for: Identification of a novel phage depolymerase against ST11 K64 carbapenem-resistant Klebsiella pneumoniae and its therapeutic potential
Source: J Bacteriol. 2025 Mar 26;207(4):e00387-24. doi: 10.1128/jb.00387-24 (PMC12004950; doi:10.1128/jb.00387-24)
Supplement: Supplemental material — Annotation of phage vB_KpnP_IME1309 sequence. [file jb.00387-24-s0001.docx]

Supplementary Table 1. Annotation of phage vB_KpnP_IME1309 sequence (accession no. PP734006.1).

| Query_name | Query_description | Query_length | Query_start | Query_end | Hit_name | Hit_description | Evalue | Identity |
| --- | --- | --- | --- | --- | --- | --- | --- | --- |
| 1 | 949-1146 + length=65 aa | 65 | 1 | 65 | UUB18478.1 | hypothetical protein | 6.56E-32 | 86.15 |
| 2 | 1396-2415 + length=339 aa | 339 | 1 | 339 | UXD79287.1 | hypothetical protein | 8.06E-241 | 97.64 |
| 3 | 2504-5224 + length=906 aa | 906 | 1 | 906 | UGC97318.1 | DNA-directed RNA polymerase | 0 | 99.12 |
| 4 | 5323-5883 + length=186 aa | 186 | 1 | 186 | YP_009816996.1 | hypothetical protein | 4.17E-119 | 96.77 |
| 5 | 5937-6146 + length=69 aa | 69 | 1 | 69 | YP_009816997.1 | hypothetical protein | 2.02E-39 | 100 |
| 6 | 6480-7550 + length=356 aa | 356 | 13 | 356 | YP_009816998.1 | DNA ligase | 9.06E-255 | 99.13 |
| 7 | 7667-7930 + length=87 aa | 87 | 1 | 87 | YP_009215465.1 | BC10 family protein DAO38970.1 MAG TPA: hypothetical protein | 4.99E-57 | 98.85 |
| 8 | 7923-8348 + length=141 aa | 141 | 1 | 141 | YP_009801401.1 | nucleotide kinase | 1.22E-96 | 97.87 |
| 9 | 8341-8493 + length=50 aa | 50 | 1 | 50 | QFR57238.1 | hypothetical protein | 2.07E-24 | 96 |
| 10 | 8571-8735 + length=54 aa | 54 | 1 | 54 | YP_009817002.1 | RNA polymerase inhibitor | 1.01E-28 | 98.15 |
| 11 | 8802-9497 + length=231 aa | 231 | 1 | 231 | YP_009796988.1 | Gp2.5-like ssDNA binding protein and ssDNA annealing protein | 2.37E-163 | 98.7 |
| 12 | 9497-9946 + length=149 aa | 149 | 1 | 149 | YP_004678737.1 | endonuclease | 4.01E-108 | 99.33 |
| 13 | 9949-10404 + length=151 aa | 151 | 1 | 151 | YP_009801321.1 | endolysin | 8.00E-108 | 98.68 |
| 14 | 10748-10993 + length=81 aa | 81 | 1 | 81 | QNN97296.1 | HNH endonuclease motif protein | 5.57E-57 | 100 |
| 15 | 11199-12806 + length=535 aa | 535 | 1 | 535 | QNN97248.1 | primase/helicase protein | 0 | 99.25 |
| 16 | 12905-13114 + length=69 aa | 69 | 1 | 69 | YP_009215474.1 | hypothetical protein | 6.27E-16 | 98.55 |
| 17 | 13114-13425 + length=103 aa | 103 | 1 | 103 | YP_009791673.1 | hypothetical protein | 2.54E-62 | 98.06 |
| 18 | 13920-16046 + length=708 aa | 708 | 1 | 708 | XAG93748.1 | DNA-directed DNA polymerase | 0 | 99.15 |
| 19 | 16372-16659 + length=95 aa | 95 | 1 | 95 | YP_009190978.1 | Gp5.5-like host HNS inhibition | 1.87E-58 | 98.95 |
| 20 | 16656-16865 + length=69 aa | 69 | 1 | 69 | YP_009190979.1 | HNS binding protein | 2.54E-42 | 98.55 |
| 21 | 16862-17068 + length=68 aa | 68 | 1 | 68 | UXD79308.1 | hypothetical protein | 2.29E-40 | 98.53 |
| 22 | 17077-17241 + length=54 aa | 54 | 1 | 54 | UXD79309.1 | hypothetical protein | 1.35E-35 | 100 |
| 23 | 17234-18139 + length=301 aa | 301 | 1 | 301 | UGC97336.1 | putative exonuclease | 3.63E-227 | 99.67 |
| 24 | 18121-18228 + length=35 aa | 35 | 1 | 35 | YP_009821364.1 | hypothetical protein | 3.21E-13 | 100 |
| 25 | 18318-18563 + length=81 aa | 81 | 1 | 81 | XAG93755.1 | hypothetical protein | 8.00E-52 | 98.77 |
| 26 | 18566-18787 + length=73 aa | 73 | 1 | 73 | WWT41899.1 | hypothetical protein | 2.00E-41 | 97.26 |
| 27 | 18789-19049 + length=86 aa | 86 | 1 | 86 | YP_002003820.1 | host range and adsorption protein | 4.15E-33 | 100 |
| 28 | 19061-20668 + length=535 aa | 535 | 1 | 535 | UTQ78107.1 | head-to-tail joining protein | 0 | 99.81 |
| 29 | 20784-21671 + length=295 aa | 295 | 1 | 295 | UXD79267.1 | hypothetical protein | 4.44E-183 | 90 |
| 30 | 21856-22890 + length=344 aa | 344 | 1 | 344 | QBG78378.1 | major capsid protein | 5.41E-237 | 99.71 |
| 31 | 23072-23650 + length=192 aa | 192 | 1 | 192 | YP_009817021.1 | tail protein | 1.58E-135 | 99.48 |
| 32 | 24113-26488 + length=791 aa | 791 | 1 | 791 | YP_009817022.1 | tail protein | 0 | 99.24 |
| 33 | 26562-26972 + length=136 aa | 136 | 1 | 136 | YP_004678758.1 | internal virion protein | 1.90E-102 | 100 |
| 34 | 26975-27565 + length=196 aa | 196 | 1 | 196 | UVX31388.1 | internal virion protein | 3.77E-120 | 96.94 |
| 35 | 27565-29820 + length=751 aa | 751 | 1 | 751 | UQK57932.1 | internal virion protein C | 0 | 98.4 |
| 36 | 29837-33787 + length=1316 aa | 1316 | 1 | 1316 | YP_009817027.1 | internal virion protein with endolysin domain | 0 | 98.94 |
| 37 | 33865-36918 + length=1017 aa | 1017 | 1 | 1017 | YP_009817028.1 | tail fiber protein | 0 | 98.82 |
| 38 | 36928-37137 + length=69 aa | 69 | 1 | 69 | CAK6605459.1 | holin | 2.00E-40 | 100 |
| 39 | 37171-37428 + length=85 aa | 85 | 1 | 85 | YP_009786841.1 | terminase small subunit | 1.92E-51 | 97.65 |
| 40 | 37868-38320 + length=150 aa | 150 | 1 | 150 | QNN97325.1 | Rz-like spanin | 2.00E-100 | 95.33 |
| 41 | 38317-40074 + length=585 aa | 585 | 1 | 585 | QOV05506.1 | terminase large subunit | 0 | 99.49 |

Supplementary Table 2. Global genome comparison of phage vB_KpnP_IME1309 with homologous phages using Blastn.

| Name of the phage | Family | Accession no. | Size (bp) | GC% | Query Cover | Per. Identity (%) | overall DNA sequence homology (%) |
| --- | --- | --- | --- | --- | --- | --- | --- |
| Klebsiella phage vB_KpnP_IME1309 | [Przondovirus](https://www.ncbi.nlm.nih.gov/Taxonomy/Browser/wwwtax.cgi?id=2570294" \o "Show taxonomy info for unclassified Przondovirus (taxid 2570294)" \t "https://blast.ncbi.nlm.nih.gov/lnktx6AFU17Z8016) | PP734006.1 | 40,829 | 52.64 | 100% | 100 | 100 |
| Klebsiella phage 175004 | [Przondovirus](https://www.ncbi.nlm.nih.gov/Taxonomy/Browser/wwwtax.cgi?id=2570294" \o "Show taxonomy info for unclassified Przondovirus (taxid 2570294)" \t "https://blast.ncbi.nlm.nih.gov/lnktx6AFU17Z8016) | PP357454.1 | 41,623 | 52.50 | 93% | 97.34 | 90.52 |
| Klebsiella phage 150007 | [Przondovirus](https://www.ncbi.nlm.nih.gov/Taxonomy/Browser/wwwtax.cgi?id=2570294" \o "Show taxonomy info for unclassified Przondovirus (taxid 2570294)" \t "https://blast.ncbi.nlm.nih.gov/lnktx6AFU17Z8016) | OP103917.1 | 42,064 | 52.50 | 93% | 96.90 | 90.11 |
| Klebsiella phage 150021 | [Przondovirus](https://www.ncbi.nlm.nih.gov/Taxonomy/Browser/wwwtax.cgi?id=2570294" \o "Show taxonomy info for unclassified Przondovirus (taxid 2570294)" \t "https://blast.ncbi.nlm.nih.gov/lnktx6AFU17Z8016) | OP103921.1 | 41,658 | 52.50 | 93% | 96.85 | 90.07 |
| Klebsiella phage 150004 | [Przondovirus](https://www.ncbi.nlm.nih.gov/Taxonomy/Browser/wwwtax.cgi?id=2570294" \o "Show taxonomy info for unclassified Przondovirus (taxid 2570294)" \t "https://blast.ncbi.nlm.nih.gov/lnktx6AFU17Z8016) | OP045496.1 | 41,643 | 52.50 | 93% | 96.94 | 90.15 |
| Klebsiella phage P06 | [Przondovirus](https://www.ncbi.nlm.nih.gov/Taxonomy/Browser/wwwtax.cgi?id=2570294" \o "Show taxonomy info for unclassified Przondovirus (taxid 2570294)" \t "https://blast.ncbi.nlm.nih.gov/lnktx6AFU17Z8016) | OR387547.1 | 40,632 | 52.50 | 93% | 95.57 | 88.88 |
| Klebsiella phage 175041 | [Przondovirus](https://www.ncbi.nlm.nih.gov/Taxonomy/Browser/wwwtax.cgi?id=2570294" \o "Show taxonomy info for unclassified Przondovirus (taxid 2570294)" \t "https://blast.ncbi.nlm.nih.gov/lnktx6AFU17Z8016) | PP357482.1 | 41,589 | 52.50 | 92% | 97.34 | 89.55 |
| Klebsiella phage 175040 | [Przondovirus](https://www.ncbi.nlm.nih.gov/Taxonomy/Browser/wwwtax.cgi?id=2570294" \o "Show taxonomy info for unclassified Przondovirus (taxid 2570294)" \t "https://blast.ncbi.nlm.nih.gov/lnktx6AFU17Z8016) | PP357481.1 | 41,589 | 52.50 | 92% | 97.34 | 89.55 |
| Klebsiella phage 175013 | [Przondovirus](https://www.ncbi.nlm.nih.gov/Taxonomy/Browser/wwwtax.cgi?id=2570294" \o "Show taxonomy info for unclassified Przondovirus (taxid 2570294)" \t "https://blast.ncbi.nlm.nih.gov/lnktx6AFU17Z8016) | PP357462.1 | 41,589 | 52.50 | 92% | 97.34 | 89.55 |
| Klebsiella phage kpssk3 | [Przondovirus](https://www.ncbi.nlm.nih.gov/Taxonomy/Browser/wwwtax.cgi?id=2570294" \o "Show taxonomy info for unclassified Przondovirus (taxid 2570294)" \t "https://blast.ncbi.nlm.nih.gov/lnktx6AFU17Z8016) | NC_048114.1 | 40,539 | 53.00 | 92% | 96.33 | 88.62 |
| Klebsiella phage 175014 | [Przondovirus](https://www.ncbi.nlm.nih.gov/Taxonomy/Browser/wwwtax.cgi?id=2570294" \o "Show taxonomy info for unclassified Przondovirus (taxid 2570294)" \t "https://blast.ncbi.nlm.nih.gov/lnktx6AFU17Z8016) | PP357463.1 | 41,589 | 52.50 | 92% | 95.62 | 87.97 |
| Klebsiella phage 175024 | [Przondovirus](https://www.ncbi.nlm.nih.gov/Taxonomy/Browser/wwwtax.cgi?id=2570294" \o "Show taxonomy info for unclassified Przondovirus (taxid 2570294)" \t "https://blast.ncbi.nlm.nih.gov/lnktx6AFU17Z8016) | PP357472.1 | 40,895 | 53.00 | 91% | 93.12 | 84.73 |
| Klebsiella phage 150031 | [Przondovirus](https://www.ncbi.nlm.nih.gov/Taxonomy/Browser/wwwtax.cgi?id=2570294" \o "Show taxonomy info for unclassified Przondovirus (taxid 2570294)" \t "https://blast.ncbi.nlm.nih.gov/lnktx6AFU17Z8016) | OP103922.1 | 41,374 | 52.50 | 91% | 95.47 | 86.87 |
| Klebsiella phage 066053 | [Przondovirus](https://www.ncbi.nlm.nih.gov/Taxonomy/Browser/wwwtax.cgi?id=2570294" \o "Show taxonomy info for unclassified Przondovirus (taxid 2570294)" \t "https://blast.ncbi.nlm.nih.gov/lnktx6AFU17Z8016) | MW042807.1 | 40,185 | 53.00 | 90% | 94.16 | 84.74 |
| Klebsiella phage ph168 | [Przondovirus](https://www.ncbi.nlm.nih.gov/Taxonomy/Browser/wwwtax.cgi?id=2570294" \o "Show taxonomy info for unclassified Przondovirus (taxid 2570294)" \t "https://blast.ncbi.nlm.nih.gov/lnktx6AFU17Z8016) | OR684907.1 | 40,222 | 53.00 | 90% | 93.86 | 84.47 |
| Klebsiella phage NL_ZS_2 | [Przondovirus](https://www.ncbi.nlm.nih.gov/Taxonomy/Browser/wwwtax.cgi?id=2570294" \o "Show taxonomy info for unclassified Przondovirus (taxid 2570294)" \t "https://blast.ncbi.nlm.nih.gov/lnktx6AFU17Z8016) | MT813141.1 | 40,222 | 53.00 | 90% | 93.85 | 84.46 |
| Klebsiella phage 175006 | [Przondovirus](https://www.ncbi.nlm.nih.gov/Taxonomy/Browser/wwwtax.cgi?id=2570294" \o "Show taxonomy info for unclassified Przondovirus (taxid 2570294)" \t "https://blast.ncbi.nlm.nih.gov/lnktx6AFU17Z8016) | PP357456.1 | 40,086 | 53.00 | 90% | 92.57 | 83.31 |
| Klebsiella phage SH-Kp 152234 | [Przondovirus](https://www.ncbi.nlm.nih.gov/Taxonomy/Browser/wwwtax.cgi?id=2570294" \o "Show taxonomy info for unclassified Przondovirus (taxid 2570294)" \t "https://blast.ncbi.nlm.nih.gov/lnktx6AFU17Z8016) | NC_047980.1 | 40,578 | 53.00 | 90% | 93.52 | 84.16 |
| Klebsiella phage 150016 | [Przondovirus](https://www.ncbi.nlm.nih.gov/Taxonomy/Browser/wwwtax.cgi?id=2570294" \o "Show taxonomy info for unclassified Przondovirus (taxid 2570294)" \t "https://blast.ncbi.nlm.nih.gov/lnktx6AFU17Z8016) | OP103920.1 | 41,215 | 52.50 | 90% | 95.52 | 85.96 |
| Klebsiella phage 150015 | [Przondovirus](https://www.ncbi.nlm.nih.gov/Taxonomy/Browser/wwwtax.cgi?id=2570294" \o "Show taxonomy info for unclassified Przondovirus (taxid 2570294)" \t "https://blast.ncbi.nlm.nih.gov/lnktx6AFU17Z8016) | OP103919.1 | 40,798 | 52.50 | 90% | 95.46 | 85.91 |
| Klebsiella phage 066056 | [Przondovirus](https://www.ncbi.nlm.nih.gov/Taxonomy/Browser/wwwtax.cgi?id=2570294" \o "Show taxonomy info for unclassified Przondovirus (taxid 2570294)" \t "https://blast.ncbi.nlm.nih.gov/lnktx6AFU17Z8016) | MW042808.1 | 40,952 | 52.50 | 90% | 95.30 | 85.77 |
| Klebsiella phage 175027 | [Przondovirus](https://www.ncbi.nlm.nih.gov/Taxonomy/Browser/wwwtax.cgi?id=2570294" \o "Show taxonomy info for unclassified Przondovirus (taxid 2570294)" \t "https://blast.ncbi.nlm.nih.gov/lnktx6AFU17Z8016) | PP357475.1 | 52,331 | 53.00 | 89% | 93.76 | 84.38 |
| Klebsiella phage 175018 | [Przondovirus](https://www.ncbi.nlm.nih.gov/Taxonomy/Browser/wwwtax.cgi?id=2570294" \o "Show taxonomy info for unclassified Przondovirus (taxid 2570294)" \t "https://blast.ncbi.nlm.nih.gov/lnktx6AFU17Z8016) | PP357467.1 | 52,172 | 53.00 | 89% | 93.83 | 84.44 |
| Klebsiella phage 175017 | [Przondovirus](https://www.ncbi.nlm.nih.gov/Taxonomy/Browser/wwwtax.cgi?id=2570294" \o "Show taxonomy info for unclassified Przondovirus (taxid 2570294)" \t "https://blast.ncbi.nlm.nih.gov/lnktx6AFU17Z8016) | PP357466.1 | 52,447 | 53.00 | 89% | 93.79 | 84.41 |
| Klebsiella phage TUN1 | [Przondovirus](https://www.ncbi.nlm.nih.gov/Taxonomy/Browser/wwwtax.cgi?id=2570294" \o "Show taxonomy info for unclassified Przondovirus (taxid 2570294)" \t "https://blast.ncbi.nlm.nih.gov/lnktx6AFU17Z8016) | HG994092.1 | 41,181 | 53.00 | 89% | 93.78 | 83.46 |
| Klebsiella phage P509 | [Przondovirus](https://www.ncbi.nlm.nih.gov/Taxonomy/Browser/wwwtax.cgi?id=2570294" \o "Show taxonomy info for unclassified Przondovirus (taxid 2570294)" \t "https://blast.ncbi.nlm.nih.gov/lnktx6AFU17Z8016) | MT542697.1 | 40,954 | 53.00 | 89% | 95.29 | 84.80 |
| Klebsiella phage RCIP0028 | [Przondovirus](https://www.ncbi.nlm.nih.gov/Taxonomy/Browser/wwwtax.cgi?id=2570294" \o "Show taxonomy info for unclassified Przondovirus (taxid 2570294)" \t "https://blast.ncbi.nlm.nih.gov/lnktx6AFU17Z8016) | OR532822.1 | 40,196 | 53.00 | 89% | 93.79 | 83.47 |
| Klebsiella phage 175029 | [Przondovirus](https://www.ncbi.nlm.nih.gov/Taxonomy/Browser/wwwtax.cgi?id=2570294" \o "Show taxonomy info for unclassified Przondovirus (taxid 2570294)" \t "https://blast.ncbi.nlm.nih.gov/lnktx6AFU17Z8016) | PP357477.1 | 40,628 | 53.00 | 89% | 93.57 | 83,27 |
| Klebsiella phage NL_ZS_1 | [Przondovirus](https://www.ncbi.nlm.nih.gov/Taxonomy/Browser/wwwtax.cgi?id=2570294" \o "Show taxonomy info for unclassified Przondovirus (taxid 2570294)" \t "https://blast.ncbi.nlm.nih.gov/lnktx6AFU17Z8016) | MT813140.1 | 40,428 | 53.00 | 89% | 93.97 | 83.63 |

*****Overall DNA sequence homolog was defined as coverage multiplied by identity, obtained after BLASTn comparison with phage vB_KpnP_IME1309 according to the International Committee on Taxonomy of Viruses (ICTV).
